# Genetic and Transcriptomic Profiles of Inflammation in Neurodegenerative Diseases: Alzheimer, Parkinson, Creutzfeldt-Jakob and Tauopathies

**DOI:** 10.3390/ijms17020206

**Published:** 2016-02-04

**Authors:** Irene López González, Paula Garcia-Esparcia, Franc Llorens, Isidre Ferrer

**Affiliations:** 1 Institute of Neuropathology, Bellvitge University Hospital, IDIBELL, Barcelona 08034, Spain; ilopez@idibell.cat (I.L.G.); pgarcia@idibell.cat (P.-G.E.); franc.llorens@gmail.com (F.L.); 2 CIBERNED (Centro de Investigación Biomédica en Red de Enfermedades Neurodegenerativas), Institute Carlos III, Madrid 28029, Spain; 3 Department of Pathology and Experimental Therapeutics, University of Barcelona, 08907 Hospitalet de Llobregat, Barcelona 08034, Spain

**Keywords:** inflammation, microglia, cytokines, complement, toll-like receptors, chemokines, Alzheimer’s disease, Parkinson’s disease, Creutzfeldt-Jakob’s disease, tauopathies

## Abstract

Polymorphisms in certain inflammatory-related genes have been identified as putative differential risk factors of neurodegenerative diseases with abnormal protein aggregates, such as sporadic Alzheimer’s disease (AD) and sporadic Parkinson’s disease (sPD). Gene expression studies of cytokines and mediators of the immune response have been made in post-mortem human brain samples in AD, sPD, sporadic Creutzfeldt-Jakob disease (sCJD) subtypes MM1 and VV2, Pick’s disease (PiD), progressive supranuclear palsy (PSP) and frontotemporal lobar degeneration linked to mutation P301L in *MAPT* Frontotemporal lobar degeneration-tau (FTLD-tau). The studies have disclosed variable gene regulation which is: (1) disease-dependent in the frontal cortex area 8 in AD, sPD, sCJD MM1 and VV2, PiD, PSP and FTLD-tau; (2) region-dependent as seen when comparing the entorhinal cortex, orbitofrontal cortex, and frontal cortex area 8 (FC) in AD; the substantia nigra, putamen, FC, and angular gyrus in PD, as well as the FC and cerebellum in sCJD; (3) genotype-dependent as seen considering sCJD MM1 and VV2; and (4) stage-dependent as seen in AD at different stages of disease progression. These observations show that regulation of inflammation is much more complicated and diverse than currently understood, and that new therapeutic approaches must be designed in order to selectively act on specific targets in particular diseases and at different time points of disease progression.

## 1. Introduction

Alzheimer’s disease (AD), Parkinson’s disease (PD), Creutzfeldt-Jakob’s disease (CJD), and tauopathies are neurodegenerative diseases presenting most commonly in aged individuals, and characterized by the accumulation of abnormal protein aggregates in the neuropil, neurons, and, in some conditions, also in glial cells, β-amyloid and hyper-phosphorylated 3R and 4R tau in AD, altered α-synuclein in PD, 3R or 4R hyper-phosphorylated tau in tauopathies, and pathogenic, mostly proteinase resistant, prion protein or prion in CJD. A percentage of cases are familial due to mutations in specific genes such as *APP* (amyloid beta (A4) precursor protein), *PSN1* (presenilin 1), and *PSN2* (presenilin 2) in AD; *PARK1/SNCA* (α-synuclein), *PARK2* (parkinson protein 2, E3 ubiquitin ligase, parkin), *PARK6* (PINK1: PTEN induced putative kinase 1), *PARK7* (DJ1), *PARK8/LRRK2* (leucine-rich repeat kinase 2), *PARK9/ATP13A2* (ATPase type 13A2), *PARK14/(PLA2G6* (phospholipase A2, group 6), *PARK15/FBX07* (F-box protein 7), *PARK17/VP35* (vacuolar protein sorting 35 homolog), and *PANK2* (pantothenate kinase 2) in PD; *MAPT* (microtubule associated protein tau) in tauopathies; and *PRNP* (prion protein) in CJD. However, the majority of cases are sporadic, although genetic modulators anticipate or regulate the phenotype of particular diseases, as does *APOE* (apolipoprotein E) in AD and *PRNP* codon 129 polymorphism in CJD [1].

Inflammation, as defined by microglial activation, and accompanied by astrocyte responses, together with increased expression of cytokines and mediators of the immune system in the brain and cerebrospinal fluid (CSF), is constant in all these neurodegenerative diseases and has been described in many studies and reviews [2,3,4,5,6,7,8,9,10].

Inflammation has pathogenic implications, either resulting from primary responses geared to reducing tissue damage secondary to toxic protein oligomers, oxidative stress, reticulum stress, and variegated molecules that might hamper neuronal viability, or leading to chronic and aggressive responses which jeopardize neuron survival. Anti-inflammatory therapies at advanced stages of the diseases have yielded poor benefits, but the use of non-steroidal anti-inflammatory drugs (NSAIDs) in younger individuals affected by autoimmune diseases such as rheumatoid arthritis has proved to be protective against AD [11,12,13,14,15,16,17,18,19] and PD [20,21].

The present review up-dates general aspects linked to neuroinflammation in AD, PD, CJD and tauopathies, and summarizes personal work published in the last two years dealing with gene expression profiles of cytokines and mediators of the immune response in a wide range of neurodegenerative diseases including AD, sPD, sCJD subtypes MM1 and VV2 at different stages of disease progression and in specific vulnerable regions. This is followed by a description of unpublished observations in the frontal cortex in tauopathies including Pick’s disease, progressive supranuclear palsy and frontotemporal lobar degeneration linked to the P301L mutation in *MAPT*. This last set of data was considered appropriate to includea here as it was obtained using the same methods and procedures as in studies noted in previous paragraphs, and it further documents disease specificities in the inflammatory response among neurodegenerative diseases with abnormal protein aggregates.

## 2. Inflammatory Genes as Risk Factors of Sporadic AD

APOEε4 augments the risk of late-onset Alzheimer’s disease (LOAD) whereas increased APOEε2 protects from AD [22,23,24,25,26,27]. In addition to other functions, APOE facilitates β-amyloid degradation in cooperative action of astrocytes and microglia [28,29,30,31,32,33]. However, the exact role of APOE isoforms as up- or down-regulators of the immune response apparently depends on the settings of experimental studies [34,35,36,37].

Variations in several genes putatively involved in inflammation and immune responses have been suggested as risk factors of LOAD on the basis of a genome-wide association study (GWAS) searching for single nucleotide polymorphisms associated with LOAD, data from the LOAD Consortium, and whole genome sequencing. These include *ABCA7* (ATP-binding cassette subfamily A member 7), *CD2AP* (CD2 associated protein), *CD33* (CD3 molecule), *CLU* (clusterin), *CRI* (complement component 3b/4b receptor 1), *EPHA1* (EPH receptor 1), *HLA-DRB5* (major histocompatibility complex, class II DR β5), *INPP5D* (inositol polyphosphate-5-phosphatase D), *PICALM* (phosphatidylinositol binding clathrin assembly protein), *PLD3* (phospholipase D family, member 3), *SORL1* (sortilin-related receptor, L (DLR class) A repeats containing), and *TREM2* (triggering receptor expressed on myeloid cells 2) [38,39,40,41,42,43,44,45,46,47,48,49,50,51,52,53,54,55,56,57,58,59,60,61,62,63].

*ABCA7*, *CLU*, and *CRI* are implicated in the complement response; *TREM2* regulates cytokine production; *HLA-DRB5*, like other molecules related to the HLA Class II, is involved in the presentation of immune-peptides; *EPHA1* and *INPP5D* participate in inflammatory responses although their exact mechanism is not known; finally, the role of *PICALM*, *PLD3*, and *SORL1* in the control of inflammation is poorly understood in AD, but PICALM seems to interact with APOE genotypes [41,64]. In addition to polymorphisms in those genes seen as putative risk factors of AD, *IL6*, *IL1B*, *IL10*, *CCL2*, *CCL3*, *SELE*, *ICAM1*, *MMP3,* and *MMP9* gene polymorphisms have been linked to sporadic AD [65,66,67,68].

## 3. Inflammatory Genes as Risk Factors of Sporadic PD

Several studies associate genetic variations in the human leukocyte antigen (HLA) region with sporadic PD [69,70,71,72]. Targeted genetic studies and GWAS also revealed that genes implicated in the regulation of leucocyte and lymphocyte activity and cytokine-mediated signalling might be risk factors of PD [73,74,75,76,77,78,79,80,81]. However, some of these observations must be approached with caution, as they may be restricted to particular populations [82].

## 4. Inflammatory Genes as Risk Factors of Tauopathies

No inflammatory genes have been reported as risk factors of progressive supranuclear palsy (PSP) to our knowledge [83]. APOEε4 has been associated with frontotemporal lobar degeneration (FTLD) in males but not in females [84]. APOEε2 has been associated with protection [85] and with higher vulnerability [86] to FTLD.

## 5. Systemic Infections as Risk Factors of AD, PD, and CJD

Recent epidemiological studies have suggested that systemic inflammation and sepsis and certain infections increase the risk of suffering from AD in old age [87,88,89,90]. Periodontal infections are a risk factor of AD [91,92] (Sparks *et al.*, 2012; Kamer *et al.*, 2015). Infectious burden of cytomegalovirus, herpes virus 1, *Borrelia burgdorferi*, *Chlamydophila pneumonia,* and *Helicobacter pylori* is associated with AD [93,94,95,96,97,98].

Similarly, a few studies in PD have suggested that certain viral infections may predispose to PD in susceptible populations [99,100]. Autoimmunity has also been implicated in the pathogenesis of sporadic PD [101,102]; the role of toxoplasmosis and influenza [103,104] requires validation.

A particular scenario focuses on the gut as the putative entry point of pathogens later affecting the nervous system. In favor of this hypothesis is the well-known transmission of several prion diseases—kuru and variant Creutzfeldt-Jabo disease (vCJD) in humans and bovine spongiform encephalopathy—following contaminated food intake. Recent studies have demonstrated the effects of the composition of intestinal microbiota in the development of the immune system and particularly of microglia [105,106,107,108]. Altered microbioma is found in PD, although we do not know at present whether its modified composition is cause or effect of PD [109].

## 6. General Aspects of Inflammation in Sporadic AD

Hundreds of studies have analysed inflammation in AD including microglial and astrocyte responses, cytokines and mediators of inflammation, and relationships between β-amyloid and different cell actors [110,111,112,113,114,115,116,117,118,119,120].

The majority of studies have been centered on the responses of microglia to β-amyloid due to the overwhelming impact of the β-amyloid cascade hypothesis on the pathogenesis of AD. Little is known, however, about regional and temporal differences of microglial reactions, and the possible relationship of inflammatory responses and hyper-phosphorylated tau deposition in NFTs.

Interestingly, neuropathological studies have stressed that neuroinflammation is an early event in the pathogenesis of AD [121].

## 7. General Aspects of Inflammation in Sporadic PD

Inflammation including activation of microglia and astrocytes, and increased expression of cytokines and variegated molecules linked to immune responses is well documented in sporadic PD [4,122,123,124,125,126,127,128,129,130,131,132]. This has been tested *in vivo* using positron emission tomography (PET) with specific ligands, in the CSF and in post-mortem brains [4,122,127,133,134,135,136,137,138]. Inflammatory responses involve the substantia nigra, putamen, and other regions such as the hippocampus [123,134,139,140,141,142,143,144,145]. All these observations point to the activation of innate CNS immune responses in sporadic PD.

In addition, lymphocytes infiltrate the substantia nigra in PD [122,146,147], and CD+4 and CD+8 T cells are recruited to the substantia nigra in PD [148].

## 8. General Aspects of Inflammation in Sporadic Creutzfeldt-Jakob Disease (sCJD)

Dramatic activation of microglia occurs in sCJD, and microglia play a cardinal role in the pathogenesis of sCJD [149,150,151,152]. The majority of studies have been focused on microglia and different patterns of activation in CJD subtypes [153,154,155,156,157,158,159,160,161,162,163,164].

Increased expression of selected pro- and anti-inflammatory cytokines and immune response mediators has been reported in the CSF and in the brain of sCJD cases [154,156,158,160,165,166,167,168,169].

## 9. General Aspects of Inflammation in Tauopathies

Only a few studies have been focused on inflammatory changes in the cerebral cortex in tauopathies. Increased numbers of microglia are found in the cerebral cortex and subcortical white matter in vulnerable cortical areas in PiD [170,171]. Microglia activation and increased expression of IL-1β has also been reported in the substantia nigra and striatum in PSP [172]. Microglial activation, as revealed with PET studies using [11C](R)-PK11195 as a marker of microglia, has been reported *in vivo* in patients with FTLD-tau [173]. Activated microglia, and increased (IL-1β), and cyclooxygenase-2 immunoreactivity has been described in one patient with FTLD linked to the P301L mutation in MAPT [174].

## 10. A Comparative Inflammatory Transcriptome Study in Neurodegenerative Diseases with Abnormal Protein Aggregates

A few years ago, we began a comprehensive study of gene expression of cytokines and mediators of the immune response in AD, PD, sCJD, and selected tauopathies, in different regions and at different stages of disease progression. mRNA expression levels of selected genes of complement system (C1QL1, C1QTNF7, C3AR1), colony stimulation factors (CSF1R, CSF3R), Toll-like receptors (TLR4, TLR7), IL8, pro-inflammatory cytokines (IL6, IL6ST, IL1B), TNF-α family (TNFRS1A, TNF-α), IL-10 family (IL10, IL10RA, IL10RB), and TGF-β family (TGFB1, TGFB2) were analysed in 20 middle-aged individuals with no neuropathological lesions, 58 age-matched controls, 105 cases of sporadic AD, 56 cases of sporadic PD, 30 cases of sCJD (15 MM1 and 15 VV2), and 18 tauopathies. Cases with associated neurodegenerative processes (*i.e.*, Lewy pathology and argyrophilic grain pathology) and metabolic syndrome were not included in the present series. Importantly, cases with systemic inflammatory (including autoimmune) diseases and infectious diseases were rejected. Special care was also taken not to include cases with prolonged agonal state (patients subjected to intensive care or suffering from hypoxia). Only cases with minor changes consistent with small blood vessel disease were included in the studies [175,176,177].

It is worth stressing that the same methods and the same probes were used in every case to minimize variations related to external conditions; taken together, these studies represent an effort to compare inflammatory transcriptomes along different neurodegenerative diseases. Methods employed are summarized as follows.

The purification of RNA was carried out with RNeasy Lipid Tissue Mini Kit (Qiagen GmbH, Hilden, Germany) following the protocol provided by the manufacturer. Quality of isolated RNA was first measured with Bioanalyzer Assay (Agilent, Santa Clara, CA, USA). The concentration of each sample was obtained from A260 measurements with Nanodrop 1000 (Thermo Scientific, Wilmington, DE, USA). RNA integrity was tested using the Agilent 2100 BioAnalyzer (Agilent Technologies, Palo Alto, CA, USA). The retro-transcriptase reaction was carried out using a High-Capacity cDNA Archive kit (Applied Biosystems, Foster City, CA, USA) following the protocol provided by the supplier. Parallel reactions for a RNA sample were run in the absence of MultiScribe Reverse Transcriptase to assess the degree of contaminating genomic DNA. TaqMan qRT-PCR assays for each gene were performed in duplicate on cDNA samples in 384-well optical plates using an ABI Prism 7900 Sequence Detection system (Applied Biosystems). For each 10 µL TaqMan reaction, 4.5 µL cDNA was mixed with 0.5 µL 20× TaqMan Gene Expression Assays and 5 µL of 2× TaqMan Universal PCR Master Mix (Applied Biosystems). Parallel assays for each sample were carried out using probes for hypoxanthine-guanine phosphoribosyltranferase (HPRT), alanyl-tRNA synthase (Aars), X-prolyl aminopeptidase (aminopeptidase P) 1 (XPNPEP1), and β-glucuronidase (β-Gus) used as housekeeping genes for normalization. The reactions were carried out using the following parameters: 50 °C for 2 min, 95 °C for 10 min, and 40 cycles of 95 °C for 15 s and 60 °C for 1 min. Finally, all TaqMan PCR data were captured using the Sequence Detection Software (SDS version 1.9, Applied Biosystems). Samples were analyzed with the double-delta cycle threshold (∆∆*C*_t_) method. ∆*C*_t_ values represent normalized target gene levels with respect to the internal control. ∆∆*C*_t_ values were calculated as the ∆*C*_t_ of each test sample minus the mean ∆*C*_t_ of the calibrator samples (WT aged 3 months or human controls) for each target gene. The fold change was determined using the equation 2^(−∆∆*C*t)^. Results were analysed with one-way ANOVA followed by Student’s *t*-test when required and checked with the Tukey method. Differences between mean values were considered statistically significant * *p* < 0.05; ** *p* <0.01; *** *p* <0.001.

The nomenclature of the probes used is found in Abbreviations at the end of the main text. The following paragraphs summarize the main observations of these studies.

## 11. mRNA Expression of Selected Immune- and Inflammatory-Related Genes sAD: Regional Differences and Disease Progression

This is a summary based on observations of López-González *et al.*, 2015 [177].

Cases and regions examined are summarized as follows: frontal cortex area 8: 11 AD stages I–II/0–A (average 74 ± 2.8 years), nine AD stages III–IV/A–B (average 79 ± 3.0 years), 20 AD stages V–VI/B–C (average 81 ± 1.6 years) and 15 middle-aged (MA) cases (average 63 ± 3 years); orbitofrontal cortex: 10 AD stages I–II/0–A (average 75 ± 3.2 years), 15 AD stages III–IV/A–B (average 80 ± 1.9 years), 6 AD stages V–VI/B–C (average 77 ± 5.0 years) and 11 middle-aged cases (average 53 ± 3.7 years); entorhinal cortex area 28: 11 AD stages I–II (average 77 ± 3.1 years), 13 AD stages III–IV (average 79 ± 1.7 years), 15 AD stages V–VI (average 81 ± 2.8 years) and seven control cases (average 53 ± 2.7 years). Middle-aged (MA) individuals had not suffered from neurologic and mental diseases and had no lesions on the post-mortem neuropathological examination. Cases with AD had no concomitant pathology (including Lewy bodies, TDP43 inclusions) excepting mild small blood vessel disease. Different groups were categorized according to the CERAD (Consortium to Establish a Registry for Alzheimer’s disease) stages of β-amyloid deposition 0, A, B and C. RIN (RNA integrity number) values ranged from 5.9 to 8.7. However, RIN values were similar among group in every particular region examined.

**Figure 1:**
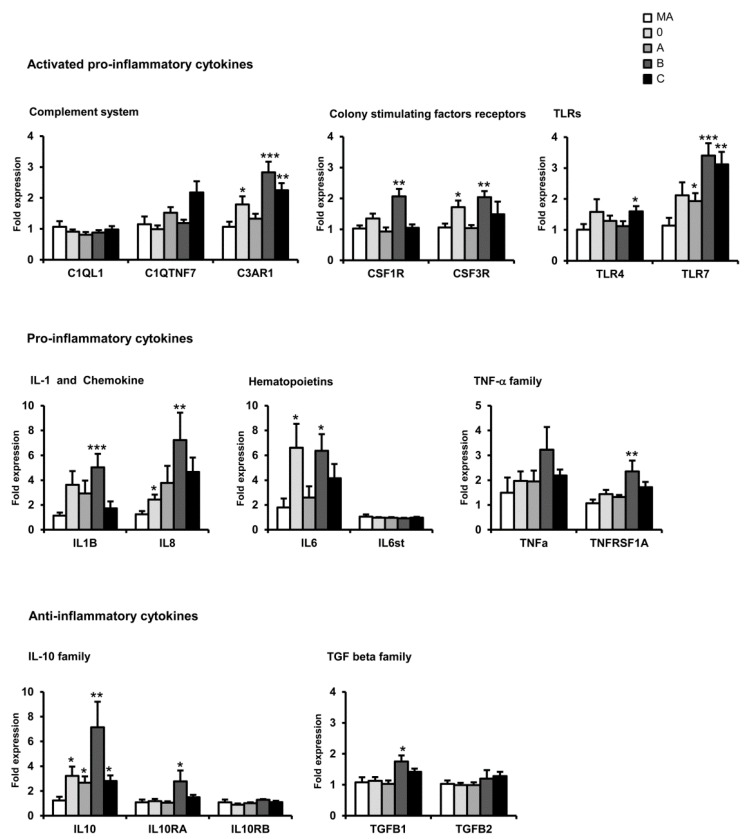
mRNA expression of selected cytokines and mediators of the immune response in the entorhinal cortex in AD cases at stages 0, A, B, and C according to the CERAD semi-quantitative assessment of neuritic plaques. Data are represented as the mean ± SEM * *p* < 0.05, ** *p* < 001, *** *p* < 0.001 (see Ref. [177] for details).

C3AR1, CSF3R, TLR7, IL1β, IL6, and IL10 mRNAs were increased in the entorhinal cortex at the first stages of AD when compared with middle-aged individuals. The expression of C1QTN7, C3AR1, TLR4, TLR7, and IL8 mRNAs significantly increased with disease progression. However, the expression of IL1β, IL6, and IL10 was reduced at advanced stages when compared with early stages of AD (Figure 1).

**Figure 2:**
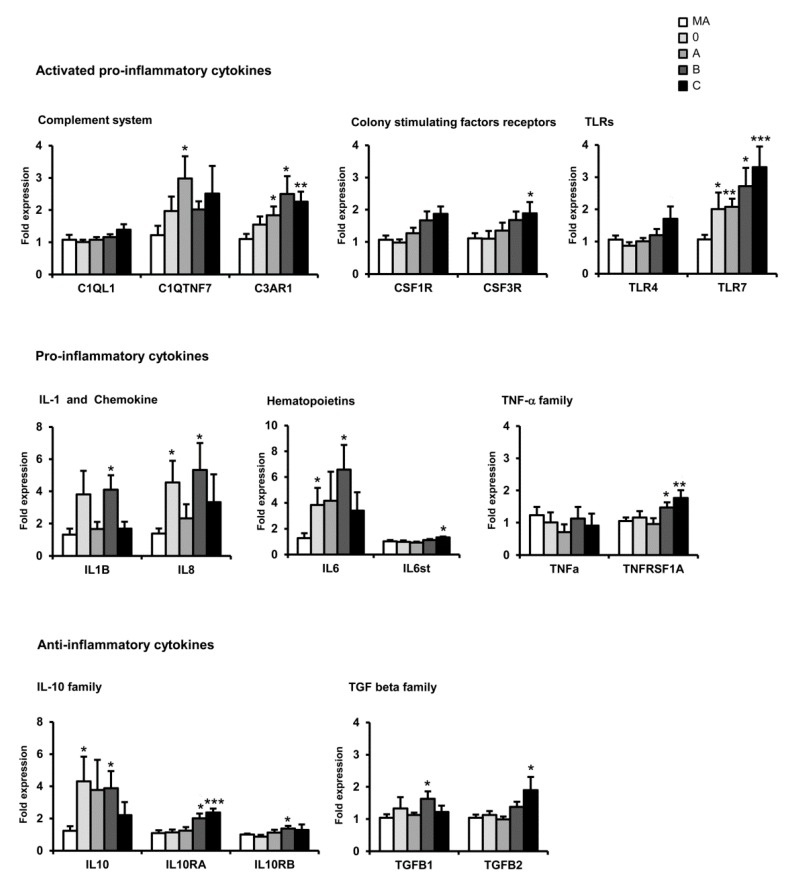
mRNA expression of selected cytokines and mediators of the immune response in the orbitofrontal cortex in AD cases at stages 0, A, B, and C according to the CERAD semi-quantitative assessment of neuritic plaques. Data are represented as the mean ± SEM * *p* < 0.05, ** *p* < 001, *** *p* < 0.001 (see Ref. [177] for details).

Up-regulation of C3AR1, TLR7, IL1β, and IL6 mRNAs was also observed in the orbitofrontal cortex at the first stages of AD when compared with MA individuals. In addition, IL10, IL8, and C1QTNF7 mRNAs were up-regulated at the first stages. Increased expression of C3AR1, CSF1R, TLR7, IL10RA, TGFβ1, and TGFβ2 mRNAs occurred with disease progression. However, C1QTNF7, C3AR1, CSF1R, TLR7, IL8, IL1β, and IL6 were down-regulated at advanced stages of the disease when compared with middle stages (Figure 2).

**Figure 3:**
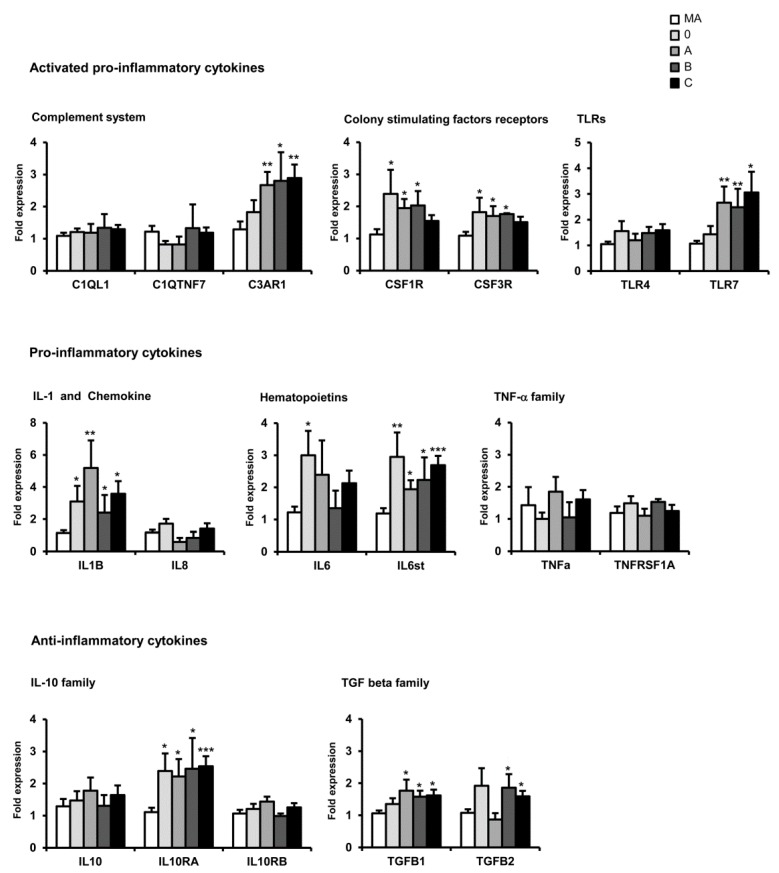
mRNA expression of selected cytokines and mediators of the immune response in the frontal cortex area 8 in AD cases at stages 0, A, B, and C according to the CERAD semi-quantitative assessment of neuritic plaques. Data are represented as the mean ± SEM * *p* < 0.05, ** *p* < 001, *** *p* < 0.001 (see Ref. [177] for details).

Significant increases in the expression levels of C3AR1, CSF1R, CSF3R, IL6, IL6ST, TGFβ1, and IL10RA were observed in the frontal cortex area 8 at the first stages of AD. mRNA expression of all these mediators increased with disease progression, as did TGFβ2. However, IL6, IL1, and CSF1R mRNA expression was reduced at advanced stages of AD (Figure 3).

The study of the mRNA expression of cytokines and mediators of the immune response in AD revealed marked regional differences among the entorhinal cortex, orbitofrontal cortex, and frontal cortex area 8 in sporadic AD. In addition, differences in the expression of such markers were also observed with disease progression; most genes were up-regulated but some of them were differentially down-regulated at advanced stages of AD.

Whether the decrease in the expression of certain mediators is related to senescence of microglia is an exciting possibility that requires further study [178,179].

## 12. mRNA Expression of Selected Immune- and Inflammatory-Related Genes in sPD: Regional Differences

This is a summary based on observations of Garcia-Esparcia *et al.*, 2014 [175].

The categorization of cases was done according to the standard classification of Braak into six stages. The total number of cases was 43 controls and 56 cases with PD-related pathology. The *substantia nigra* was studied in 14 PD cases stages 1–2 (*n* = 4), 3 (*n* = 1), 4 (*n* = 3) and 5 (*n* = 6), and 12 controls; the putamen in seven PD cases stages 3 (*n* = 1), 4 (*n* = 4) and 5 (*n* = 2), and eight controls; the frontal cortex (area 8) in 26 PD cases stages 3 (*n* = 1), 4 (*n* = 14), 5 (*n* = 10) and 6 (*n* = 1), and 21 controls; and the angular gyrus (area 39) in 13 PD cases stages 3 (*n* = 1), 4 (*n* = 8) and 5 (*n* = 4), and nine controls. Representation of genders was as follows: controls: 27 males and 16 females; PD cases: 40 males and 16 females [175]. PD cases had not any associated pathology excepting small blood vessel disease. RIN values were from 6.6 to 8.8.

Decreased expression of C1QL1, C1QTNF7, C3AR1, TLR7, IL1B, IL6, IL6ST, TNF-α, IL10, IL10RB, and TGFB2 mRNAs was observed in the substantia nigra at Braak stages 3-6. Decreased expression of C3AR1, TLR7, and TNFα, and increased expression of C1QTNF7 and IL10RA mRNAs, was observed in the putamen; down-regulation of CSF3R and TLR4, and up-regulation of IL10RA, were seen in the frontal cortex (area 8); and up-regulation of C3AR1, CSF3R, IL10RA, and TGB2 mRNAs was observed in the angular cortex (area 39) in the same series of PD cases.

The results of the study of the mRNA expression profile of cytokines and mediators of the immune response in sporadic PD showed marked regional variations, with expression levels of most genes markedly reduced in the substantia nigra pars compacta, small numbers down-regulated or up-regulated in the putamen and frontal cortex area 8, and a few up-regulated genes in the angular gyrus in the same series of cases.

## 13. mRNA Expression of Selected Immune- and Inflammatory-Related Genes in CJD: Regional Differences Depending on the Genotype

This is a summary based on observations of Llorens *et al.*, 2014 [176].

Gene expression of selected cytokines and mediators of the immune response was analyzed in the frontal cortex area 8 and cerebellum of Creutzfeldt-Jakob disease (CJD) cases subtypes MM1 (*n* = 15) and VV2 (*n* = 15), and age-matched controls (*n* = 15).

Fourteen of eighteen mRNAs of the complement system, colony stimulating factors, toll-like receptors, and pro-inflammatory and anti-inflammatory cytokines were up-regulated in the frontal cortex area 8 in sCJD MM1. However, only six mRNAs of the fourteen increased in sCJD MM1 (ILRB, TNFRSF1A, C3AR1, CSF1R and CSF3R) and were up-regulated in the frontal cortex in sCJD VV2.

Regarding the cerebellum, 16 of eighteen mRNAs were up-regulated in the cerebellum in the same sCJD VV2 series of cases, whereas only eleven were up-regulated in the cerebellum in sCJD MM1. In addition, values were higher for sCJD VV2 when compared with sCJD MM1. Curiously IL8 and TLR4 were up-regulated in the cerebellum in sCJD MM1 but not in sCJD VV2.

These observations show regional gene regulation differences in sCJD which depend on the genotype being higher in the frontal cortex in sCJD MM1 and in the cerebellum in sCJD VV2.

## 14. mRNA Expression of Selected Immune- and Inflammatory-Related Genes in Tauopathies: Disease Differences

Post-mortem samples of the frontal cortex area 8 of five cases with Pick’s disease, eight cases with progressive supranuclear palsy typical form, and five cases with frontotemporal lobar degeneration linked to mutations in *MAPT* (FTLD-tau P301L) were analyzed in parallel with frontal cortex area 8 of age-matched controls. Pathological cases were pure forms with no associated pathologies. Methods were the same as those described in previous paragraphs and utilized in the study of AD, sPD and sCJD.

**Figure 4:**
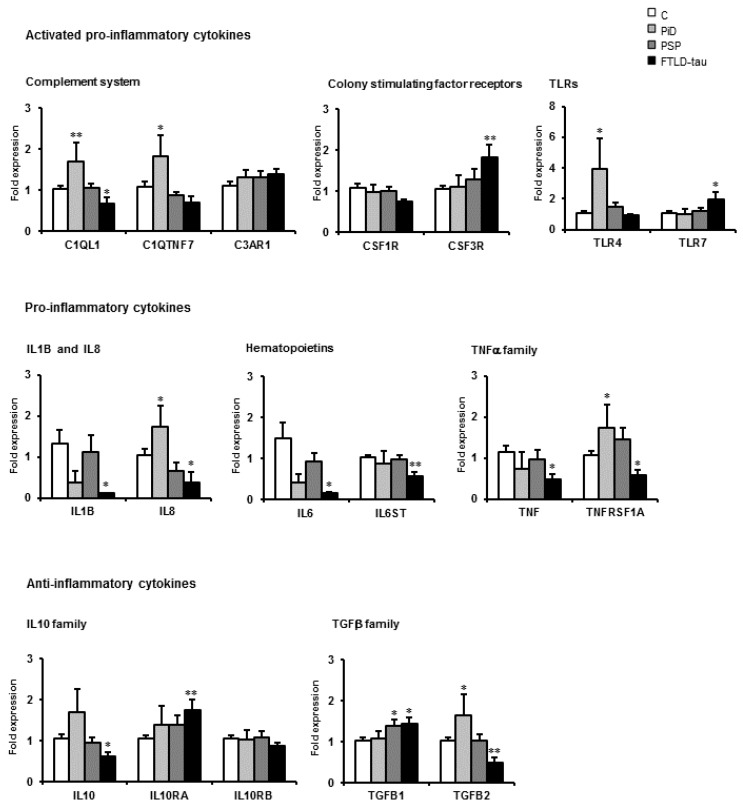
mRNA expression of selected cytokines and mediators of the immune response in the frontal cortex in Pick’s disease (PiD, *n* = 5), progressive supranuclear palsy (PSP, *n* = 8), frontotemporal lobar degeneration-tau P301L (FTLD-tau; *n* = 5), and age-matched controls (C, *n* = 18). Data are represented as the mean ± SEM * *p* < 0.05, ** *p* < 001.

Gene expression of cytokines and inflammatory-related molecules was not homogeneous among the three conditions. Major changes were seen in PiD; C1QL1, C1QTNF7, TLR4, IL8, TNFRSF1A, and TGFB2 mRNA levels were significantly increased. Not a single mRNA of the eighteen examined was de-regulated in PSP. However, C1QL1, IL1B, IL8, IL6, IL6ST, TNF, TNFRSF1A, IL10, and TGFB2 were down-regulated, and CSF3R, TLR7, and ILRA10 were up-regulated in five FTLD-tau cases bearing the P301L mutation (Figure 4).

## 15. Comparative Aspects of Inflammatory Gene Regulation in the Frontal Cortex in Neurodegenerative Diseases with Abnormal Protein Aggregates

Inflammatory gene regulation differs greatly from one condition to another not only along disease progression (as seen in AD) but also at terminal stages. The most dramatic changes, manifested as up-regulation of the vast majority of genes assessed, occur in sCJD, and these are even greater in sCJD when compared with sCJD VV2 (although the opposite occurs in the cerebellum in the same cases). Gene up-regulation in AD follows in intensity, although up-regulation is higher at early and middle stages than advanced stages of the disease. PiD shows moderate inflammatory gene up-regulation. The cerebral cortex in PD, including a few cases with dementia, exhibits limited de-regulation of cytokines and inflammatory genes. Curiously, mRNA expression levels of assessed genes in PSP did not differ from those seen in age-matched controls. Finally, FTLD-tau P301L shows down-regulation of several inflammation-related genes and increased mRNA expression of only two.

It can be argued that regional differences in the regulation of genes involved in neuroinflammation can be expected in the same brain based on the different regional neuronal vulnerability. The present observations prove that this intuitive thinking is true but also, and more importantly, that certain genes can be up-regulated in certain regions whilst down-regulated in others in the same individual.

Similarly, it can be argued that inflammatory responses can differ in distinct diseases. Again the present review proves that available data support the concept that this is true even when considering a particular brain region. Inflammatory gene regulation in the frontal cortex in the different diseases and different stages of AD is summarized in Table 1. This table clearly shows that, in a given region, inflammatory responses are disease-dependent and stage-dependent. Moreover, the patterns of gene regulation characterize inflammatory changes in particular diseases which are independent of the degree of neuronal loss. For example marked neuronal loss is observed in the frontal cortex in Pick’s disease, FTLD-tau, AD and CJD but only diseases with extracellular deposits of abnormal proteins as β-amyloid and PrP^Sc^ in AD and CJD, respectively, are accompanied by marked inflammatory responses. In contrast, tauopathies are not.

**Table 1:** Summary of gene regulation in the frontal cortex are 8 in Alzheimer’s disease (AD) at stages A.; B and C.; Parkinson’s disease (PD); Creutzfeldt-Jakob disease (CJD) subtypes MM1 and VV2.; Pick’s disease (PiD).; progressive supranuclear palsy (PSP).; and frontotemporal lobar degeneration linked to mutation P301L in MAPT (FTLD-tau).

Family	Frontal Cortex Area 8									
	AD A		AD B	AD C	PD	CJD MM1	CJD VV2	PiD	PSP	FTLD-tau
Complement system	C1QL1	=	=	=	=	=	=	↑	=	↓
	C1QTNF7	=	=	=	=	=	=	=	=	=
	C3AR1	↑	↑	↑	=	↑	↑	=	=	=
Colony stimulating factors	CSF1R	↑	↑	=	=	↑	↑	=	=	=
	CSF3R	↑	↑	=	↓	↑	↑	↑	=	↑
Toll-like receptors	TLR4	=	=	=	↓	↑	=	=	=	
	TLR7	↑	↑	↑	=	↑	=	=	=	↑
Cytokines	IL8	=	=	=	=	=	=	↑	=	↓
	IL1B	↑	↑	↑	=	=	↑	=	=	↓
	IL6	↑	=	=	=	↑	=	=	=	↓
	IL6ST	↑	↑	↑	=	↑	=	=	=	↓
TNF family	TNFα	=	=	=	=	↑	↑	=	=	↓
	TNFRS1A	=	=	=	=	↑	↑	↑	=	↓
IL10 family	IL10	=	=	=	=	↑	=	=	=	↓
	L10RA	↑	↑	↑	↑	↑	=	=	=	↑
	L10RB	=	=	=	=	=	=	=	=	=
TGF-β	-	↑	↑	↑	=	↑	=	=	↑	↑
	-	=	↑	↑	=	↑	=	↑	=	↓

## 16. Increased Expression of Inflammatory Markers in Blood and Serum in AD and PD

Several studies have shown inconsistent alterations of cytokines and mediators of the immune response in patients with mild cognitive impairment and AD [180,181,182,183,184,185,186]. Increased serum TNFR1 levels have been interpreted as a risk factor for converting mild cognitive impairment into dementia [187]. Acute systemic inflammatory disease seems to be accompanied by an increase in serum TNF-α levels and by a two-fold increase in cognitive decline in AD [88]. Whether these changes are a cause or a consequence of the disease is not known [188].

Interestingly, increased protein levels of IL-13, TNF-α, and G-CSF have been found in serum of patients with rapid AD compared to AD patients with common long progression [169].

Several studies have examined peripheral blood lymphocytes in PD [189]. Peripheral lymphocytes in PD show increased expression of cytokines compared to lymphocytes in controls when exposed to lipopolysaccharide (LPS) [190]. Examination of peripheral blood lymphocytes has shown altered percentages of T and B cells [191]; and increased CD45RO+ and FAS+ CD4+ T cells, along with decreased CD31+ and α4β7+ CD4+ T [192]. In addition, increased numbers of monocytes and elevated levels of certain cytokines such as IL-1β, IL-2, IL-4, IL-6, IL-8, IL-10, IL-12, NT-pro-2′,3′-cyclic nucleotide 3′ phosphodiesterase (NT-proCNP), tumour necrosis factor (TNF-α), soluble tumour necrosis factor α receptor-1 (TNFR1), and chemokine (C-C motif) ligand 5 (RANTES) have been reported in PD [190,193,194,195,196,197,198,199,200,201]. TNF-α expression levels in blood correlate with altered cognition, depression, and sleep disturbances in PD [201]. Other studies using microarrays have shown a limited repertoire of altered cytokines in patients with Parkinsonism [202].

## 17. Conclusions

The present observations indicate that inflammatory genes are not homogeneous risk factors in neurodegenerative diseases with abnormal protein aggregates. Available information indicates that systemic infections might be implicated in the pathogenesis of certain neurodegenerative diseases and that the peripheral immune system seems to be variably involved in the pathological processes, as revealed by subtle changes in certain cell components of blood and serum.

More importantly in the present context is the fact that regulation of genes encoding cytokines and mediators of the immune response differ from one disease to another. Regarding a particular region, the frontal cortex area 8, dramatic changes occur in sCJD subtypes MM1 and VV2, followed by sporadic AD and PiD. Gene expression alterations in the frontal cortex in sporadic PD are moderate, whereas practically no modifications are found in PSP. Interestingly, in contrast with other entities, down-regulation of several genes instead of up-regulation occurs in the frontal cortex area 8 in FTLD-tau P301L.

Another important conclusion is that regional differences occur in the same individuals. For example, the majority of assessed inflammation-related genes are down-regulated in the substantia nigra in sporadic PD whereas only a few of them are down-regulated in the putamen; down- and up-regulated genes are found in the fontal cortex area 8 while only up-regulated genes are found in the angular gyrus. Regional differences also apply in sCJD where gene expression markedly differs in the frontal cortex area 8 when compared with the cerebellum. Moreover, differences also exist between subtypes sCJD MM1 and sCJD VV2.

Finally, gene expression of cytokines and mediators of the immune response vary with disease progression, as seen at different stages of sporadic AD. Major down-regulation occurs at the first and middle stages of the disease, only to decrease at advanced stages of AD.

The present study has not looked for the possible causes of such differences, although some insights link inflammatory responses with extracellular abnormal proteins such as β-amyloid and PrP^Sc^ in AD and CJD, but rather has sought to describe neuroinflammation, inflammation, and inflammatory response in neurodegenerative diseases.

The major practical implication is that we are starting to learn that neuroinflammation is a very wide and ambiguous term when applied to neurodegenerative diseases as it covers many different responses. Neuroinflammatory responses are disparate among neurodegenerative diseases and they also differ in different regions in the same individual, and at different stages of disease progression. Knowledge of these descriptive data is necessary in order to understand the very complex scenario caused by neuroinflammation in the context of neurodegenerative diseases. A better understanding of these aspects will help to identify targets at the appropriate stages of particular diseases for new selective therapeutic interventions.

## Abbreviations

C1QL1	complement component 1, q subcomponent-like 1
C1QTNF7	C1q and tumor necrosis factor related protein 7
C3AR1	complement component 3a receptor 1
CLEC7A	C-type lectin domain family 7, member A
CSF1R	colony stimulating factor 1 receptor
CSF3R	colony stimulating factor 3 receptor
IL1B	interleukin 1β
IL6	interleukin 6
IL6ST	interleukin 6 signal transducer
IL8	interleukin 8
IL10	interleukin 10
IL10RA	interleukin 10 receptor α
IL10RB	inerkeukin 10 receptor β
NFκB	nuclear factor of kappa light polypeptide gene enhancer in B-cells
TGFB1	transforming growth factor β1
TGFB2	transforming growth factor β2
TLR4	toll-like receptor 4
TLR7	toll-like receptor 7
TNF, TNFα	tumor necrosis factor α
TNFRSF1A	tumor necrosis factor receptor superfamily, member 1A
